# Inducing Lucid Dreaming Based on a Contemplative Practice of Compassion

**DOI:** 10.3390/brainsci16030315

**Published:** 2026-03-16

**Authors:** Daniel J. Morris, Susana G. Torres-Platas, Karen R. Konkoly, John Hirschle, Lodoe Sangpo, Thabkhe, Tenzin Legden, Lobsang Pelmo, Tenzin Pasang, Marcia Grabowecky, Robin Nusslock, Ken A. Paller

**Affiliations:** 1 Department of Psychology, Northwestern University, Evanston, IL 60201, USA; danielmorris2027@u.northwestern.edu (D.J.M.); gabriela.torresplatas@northwestern.edu (S.G.T.-P.); karen@dust.systems (K.R.K.); jackhirschle2027@u.northwestern.edu (J.H.); grabowecky@northwestern.edu (M.G.); nusslock@northwestern.edu (R.N.); 2 Gaden Jangtse Monastery, Mundgod 581411, India; 3 Sera Jey Monastery, Bylakuppe 571104, India; 4 Drepung Loseling Monastery, Mundgod 581411, India; 5 Jangchub Choeling Nunnery, Mundgod 581411, India; 6 The Dalai Lama Institute for Higher Education, Bengaluru 560072, India

**Keywords:** lucid dreaming, dream yoga, contemplative sleep practices, dreaming, REM sleep

## Abstract

Background/Objectives: Lucid dreaming—dreaming with the awareness that one is dreaming—has been explored from many perspectives, including those of cognitive neuroscience and various ancient cultural traditions. Lucid dreaming appears within the Tibetan-Buddhist literature together with dream yoga, a set of contemplative practices aimed at cultivating lucidity during dreams along with other qualities such as visual imagination, somatic awareness, and cognitive flexibility. These practices include deity visualization, which is the practice of bringing to mind a detailed image of a being whose qualities the practitioner wishes to cultivate. We examined whether it is possible to induce a lucid dream of Chenrezig, the ultimate embodiment of compassion in a Tibetan-Buddhist context. Methods: Five participants slept in the sleep laboratory for 7 overnight sessions with polysomnographic recording and auditory reminders to visualize Chenrezig during REM sleep. Results: Lucid dreams were reported by two participants. A frequent lucid dreamer with no prior Tibetan-Buddhist training experienced a lucid dream that included a visualization of Chenrezig following auditory cueing during REM sleep. A monastic participant with no prior history of lucid dreaming reported their first-ever lucid dream on the night following their laboratory session. Conclusions: This exploratory study illustrates, via collaborative research including monastic scholars trained in neuroscience, that dream content can be intentionally shaped using an approach that integrates contemplative visualization practices with modern techniques of dream engineering.

## 1. Introduction

Compassion is a powerful motivator of prosocial behavior and is known to enhance emotional well-being. Contemplative practices, such as loving-kindness meditation, aim to cultivate compassion and have been shown to increase mindfulness, purpose in life, social connection, and psychological well-being [1]. According to the broaden-and-build theory, repeated experiences of positive emotions can accumulate over time to strengthen empathy and compassion [2]. This theory suggests that compassion is a trainable quality during wakefulness. However, it remains unknown whether compassion can also be intentionally cultivated during sleep, a question that motivates the present study. As a first step toward investigating the cultivation of compassion during sleep, this proof-of-concept study explores a methodology whereby compassion-related imagery can be incorporated into lucid dreams.

Tibetan dream yoga offers a framework for conceptualizing contemplative practices to cultivate lucidity, visual imagination, somatic awareness [3,4], and cognitive flexibility during dreams [5]. Cognitive flexibility is defined as the ability to appropriately adjust one’s behavior according to a changing environment [6]. Within the context of dream yoga, cognitive flexibility emphasizes one’s ability to navigate changing dream scenarios, switching them at will, and responding adaptively within the dream context. Dream yoga encompasses a set of techniques, including becoming lucid in one’s dreams, transforming dream content through practices such as conjuring, multiplying, traversing, and flying, and then recognizing the dreamlike nature of all experience. One particularly advanced practice involves conjuring a deity within the dream—and potentially transforming into that deity—a practice intended to help cultivate the qualities associated with that deity. To date, this specific practice has not been empirically studied using neuroscientific methods, despite the centrality of visualization practices within tantric Buddhist traditions [7,8]. This study lays the groundwork for future research into how such practices may shape traits like compassion, emotional regulation, or personal identity. Furthermore, this approach bridges cognitive neuroscience with Tibetan contemplative traditions and opens new avenues for understanding dream states for spiritual cultivation, transformation, and scientific inquiry [3,4,9].

Cognitive neuroscience provides tools for studying contemplative practices and lucid dreaming. Prior research has found that pre-sleep experiences and stimulation during sleep can influence dream content [10,11,12,13,14,15,16,17,18,19,20,21,22]. Lucid dreams during rapid eye movement (REM) sleep have been associated with activation in frontoparietal brain regions implicated in executive control and metacognitive processing [23]. EEG studies have further identified global reductions in delta and beta power, along with increases in signal complexity and eye-movement density—markers consistent with increased cortical and subcortical activation during lucid states [24,25,26]. A recent study analyzed scalp EEG recordings from multiple laboratories, comparing lucid and non-lucid REM sleep using source-localization methods applied to pooled datasets [27]. However, because such anatomical inferences are derived from inverse modeling of scalp EEG data, they should be interpreted cautiously. Nonetheless, the approach of contrasting electrophysiological activity between lucid and non-lucid REM sleep provides a promising framework for probing the neural correlates of lucidity.

These findings suggest that lucid dreaming engages distributed brain networks potentially involved in metacognition, imagery, and self-awareness—capacities that are also central to Tibetan Buddhist contemplative training. Yet most neuroscientific studies of lucid dreaming have focused on western populations and secular induction techniques. This project offers a unique contribution by examining lucid dreaming within a Tibetan-Buddhist framework, guided by experienced monastic scholars with extensive training within the tradition. This study did not attempt to replicate previous neurophysiological analyses of lucid dreaming; instead, electrophysiological recordings were used to assess sleep stages with procedures that included volitional signaling during lucid dreams.

Our investigation of contemplative sleep practices was founded on cross-cultural collaboration. Such collaborations have already produced many interesting findings, including initial descriptions of the neural correlates of long-term meditation practice [28,29]. One important development has been the inclusion of science education into the advanced monastic curriculum. Initiatives such as the Emory-Tibet Science Initiative (ETSI), Science for Monks and Nuns, and the Mind and Life Dialogues have supported this effort by providing scientific training for Tibetan monks and nuns. ETSI has played a unique role in developing a systematic and comprehensive science education program that has now been integrated into the core curriculum of monastic learning centers. This program now includes both research and pedagogy training offered to advanced monastic scholars [30]. One goal of Tibetan monastics studying science is to integrate modern scientific knowledge with Buddhist contemplative traditions in a way that enriches both systems. This approach supports cultural preservation, advances understanding of the mind and reality, and prepares monastics to become effective science educators and researchers within their own communities [31,32]. These programs aim not only to teach science to monastics, but to empower them to lead scientific research grounded in their contemplative context.

**Figure 1:**
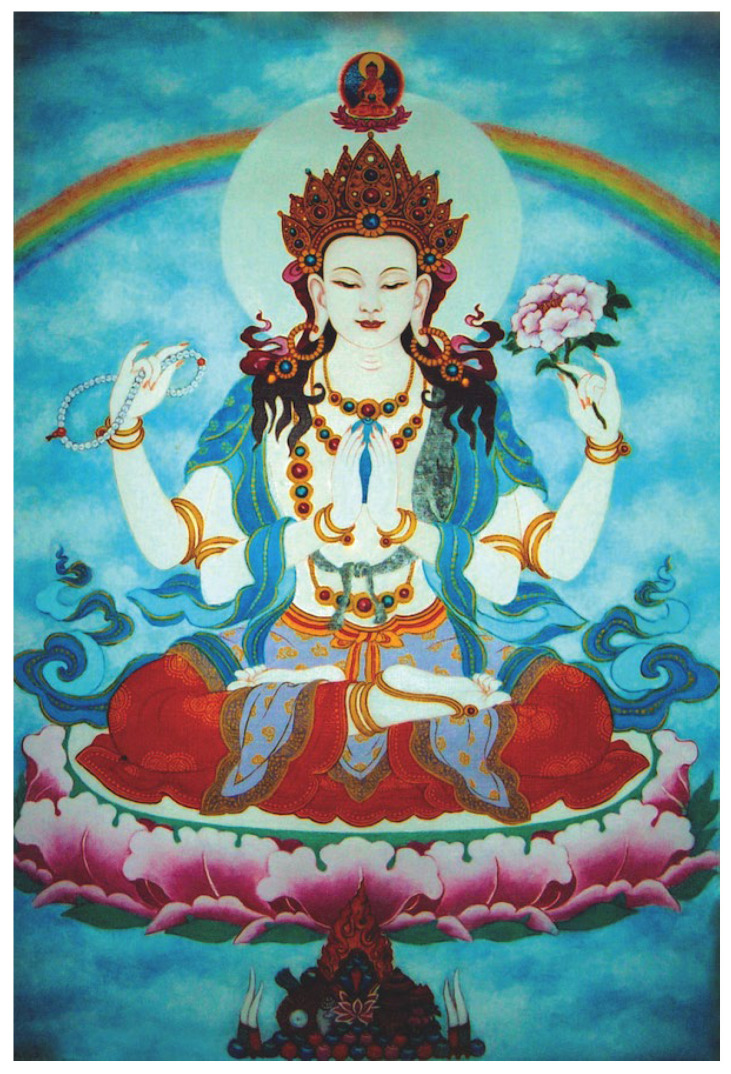
This image of Chenrezig, the Tibetan-Buddhist deity of compassion, was viewed by participants before sleep. Painting by Huei-Shion Tsai. Reproduced from *Chenrezig* *Sadhana & Commentary* (p. 3), translated by P. G. White under the guidance of Shamar Rinpoché, 2012, Bodhi Path Publishing [33]. Published with permission.

The present investigation arose as part of this scientific training for monastic scholars. In 2022, a cohort of five Tibetan scholars, including three monks, one nun, and one lay science educator, participated in the first ETSI Northwestern Neuroscience Research Internship, a three-month residency focused on methods in cognitive neuroscience. During the internship, these scholars worked closely with faculty and graduate student mentors to design and carry out a study that combined both pedagogical and scientific aims. The procedure converged with the monastic scholars’ long-standing contemplative and scholarly training, which includes Chenrezig, the ultimate embodiment of compassion in a Tibetan-Buddhist context (Figure 1).

We aimed to determine whether it is possible to intentionally induce a lucid dream about Chenrezig. The monastic scholars were not only participants and cultural informants, they also carried out many steps of the scientific process. A rich collaborative environment for scientific training was fundamental to formulating the core hypotheses and developing the experimental protocol. The monastic scholars conducted the overnight EEG data collection and participated fully in analysis and interpretation. Their authorship on the study affirms their central intellectual contribution and reflects a growing movement of monastic scholars leading scientific investigations.

## 2. Materials and Methods

### 2.1. Participants

This study began with a development phase during which four Tibetan monastic scholars on the research team participated in the study to test the protocol and refine the research design. These monastic researchers chose to participate in the overnight recordings as part of their didactic training in cognitive neuroscience. One monk and one nun each completed two sessions, resulting in a total of six overnight recordings in the development phase. The monastic participants had extensive prior training in analytic meditation, philosophical debate, and Buddhist textual study within the Gelugpa tradition of Tibetan Buddhism. Their knowledge about dream yoga was largely conceptual, and they reported that they seldom experienced lucid dreaming. After finalizing the design, the protocol was tested with one western male frequent lucid dreamer. This participant was chosen to maximize the likelihood of observing a lucid dream. He reported lucid dreams more than once per month but had no prior experience with contemplative practices like visualizing Chenrezig. Participation was voluntary, conducted under Northwestern University IRB approval, and all participants provided informed consent and received $80 compensation per night.

### 2.2. Experimental Procedure

Participants arrived at the lab 3 h before their standard bedtime. Participants were prepared for polysomnography using a NeuroScan SynAmps system (Compumedics Neuroscan, Charlotte, NC, USA) with a 1000 Hz sampling rate, which included electroencephalography (EEG) channels F3, F4, C3, C4, O1, O2, two chin electromyography (EMG) electrodes, one vertical and two horizontal electrooculography (EOG) electrodes, two electrocardiogram (ECG) electrodes, and a nasal cannula to measure airflow. Sound intensities for two auditory stimuli were determined before sleep by playing each from a speaker at increasing volumes until the participant said they could hear them and then increasing the volume until they said it was comfortable. The first sound consisted of three pure-tone beeps increasing in pitch (400, 600, and 800 Hz) at approximately 40–45 dB SPL and lasting approximately 650 ms. The second sound was the spoken word “Chenrezig.” Before sleeping, participants were shown an image of Chenrezig (Figure 1). Participants practiced meditating on Chenrezig for 15 min, including visualizing the deity’s appearance, colors, and bringing to mind the feeling of compassion embodied by Chenrezig. Participants were told that they would sleep uninterrupted for 5 h before being awoken to be trained to associate the beeping cue with becoming lucid in their dream. This method is known as Targeted Lucidity Reactivation (TLR) [34,35] and has been shown to promote lucid dreaming when combined with the wake-back-to-bed method [36,37]. Participants were awakened around 4–5 AM for the training. Participants remained awake for approximately 30 min, during which they completed the TLR training. This training included reviewing the beeping cue, practicing associating the cue with becoming lucid, and setting the intention to summon Chenrezig in their dreams. When participants entered REM sleep, they were first cued with the 3-beep sound to encourage lucidity, as well as the Chenrezig cue subsequently. The decision to combine multiple induction elements was intended to increase the likelihood of lucid dreaming; admittedly, this approach limits the ability to determine which elements contributed to lucidity and dream-content shaping. Cues were paused if any signs of arousal were detected (e.g., increased alpha level, increased muscle tone, movement). Dream reports were collected at the end of each REM period with the following questions. (i) Can you tell me everything you can remember? (ii) Did any of your experiences during sleep relate to the sounds or task? (iii) Do you remember anything else? (iv) Please try to recount in as much detail as possible the sounds you heard and signals you completed.

### 2.3. Lucid Dream Task

Participants were instructed to signal lucidity using a pre-specified horizontal eye-movement sequence (left–right–left–right) detectable in the EOG. To make the signal, the dreamer was to move their eyes as far left as possible, then as far right as possible, and then repeat those two movements (i.e., LRLR). They were instructed to clearly bring an image of Chenrezig to mind once they attained lucidity. Task progress was indicated using sniffing signals measured via the nasal cannula airflow channel: two in–out sniffs to signal attempting to summon Chenrezig, and four in–out sniffs to signal successfully summoning Chenrezig. This procedure was based on previous research showing that sniffs during lucid dreams can be clearly detected and often are less distracting for the dreamer compared to repeatedly making eye movements [5,38,39].

## 3. Results

**Figure 2:**
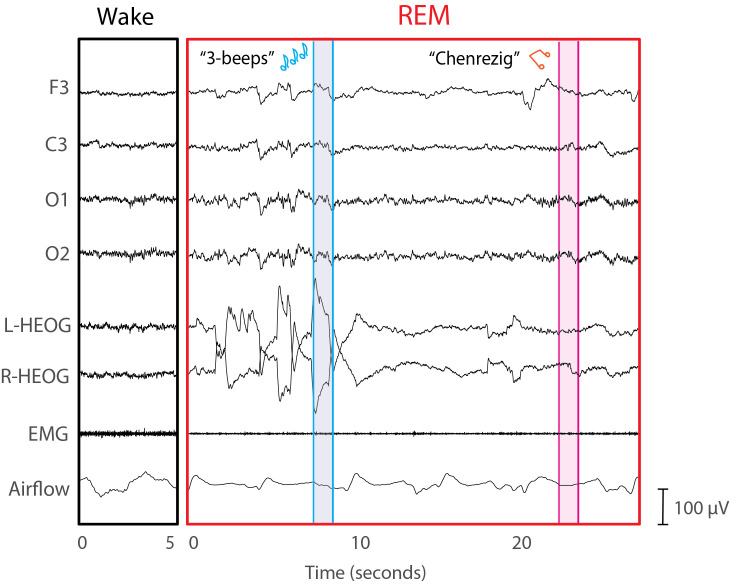
Sleep physiology during a 5 s period of wake (**left**) and a 30 s period of REM (**right**) demonstrates typical characteristics of REM sleep during the latter period. The blue-highlighted section shows when the “3-beeps” stimulus was presented to help induce lucidity, and the pink-highlighted section shows when the spoken stimulus “Chenrezig” was presented as a reminder to conjure Chenrezig. A total of twelve 3-beep cues were presented during this REM period prior to the single Chenrezig cue. The participant awakened 10 s after the Chenrezig cue and reported the following dream report: “I was with my clarinet teacher and there was a hole in my clarinet. Then, I realized I was dreaming and thought about Chenrezig. I managed to stay in the dream and the atmosphere changed to a beach right next to a city. I was by the water, and the entire dream had an untextured feel. The water was speaking to me, and it was talking about Chenrezig. I forget exactly what it was saying. Then a tsunami wave hit, and I ran back up to the shore and heard the Chenrezig sound. I tried to visualize Chenrezig there with me in the flesh, but all I saw was an outline. He was appearing like a retinal burn. But he didn’t move or say anything from what I can remember. And that was when I woke up and realized that I forgot to signal during the lucid dream.”

A lucid dream was reported by the western frequent lucid dreamer during his overnight session. EEG data and the dream report are shown in Figure 2. This participant reported lucid dreams previously described in several other papers [38,40,41]. No other participants reported having lucid dreams during their overnight sessions. Table 1 shows the number of cues presented to each participant during REM sleep. Cues were presented during every REM period after the 4 AM wake-back-to-bed procedure, and dream reports were collected after every cued REM period.

One monastic participant volunteered that they experienced their first-ever lucid dream on the night following their laboratory session. This participant reported the following dream account:

“I was with my sister, and we were on a mountain collecting firewood. Suddenly, many armies began approaching us from different directions, coming to kill us. At that moment, my other sister was shouting for us to run. But I told my sister it wasn’t a good idea to run, because the armies were positioned above us, and it would be easy for them to attack from there. I suggested we find a place to hide. As we searched for a hiding spot, my sister became increasingly anxious and afraid. I tried to calm her, saying, ‘Don’t worry—this is just a dream.’ I was aware that it wasn’t real. I wanted to carry that awareness into my meditation, but the moment I tried, I woke up.”

## 4. Discussion

This proof-of-concept study supports the feasibility of inducing a lucid dream about the Buddhist deity of compassion under controlled laboratory conditions. Although only one lucid dream was reported in the laboratory, the results illustrate how dream content can be directed towards summoning a deity. The protocol included a combination of intention-setting, pre-sleep visualization, and auditory cueing during REM sleep. We cannot infer which of these (or other) factors was most critical for producing the desired result.

The single successful example recorded in the lab with standard polysomnographic methods was with a frequent lucid dreamer who had no prior experience visualizing Chenrezig. Although this participant did not signal lucidity or task performance during sleep, his retrospective dream report was collected immediately following the REM period of interest. He reported hearing the auditory “Chenrezig” cue during the dream followed by a visual experience of the deity. The “Chenrezig” auditory cue was presented only once during this REM period, providing support for the temporal alignment of the EEG data and the reported content.

This finding suggests that deity imagery can emerge in dreams even without deep personal familiarity. This result may be especially informative for non-expert practitioners, suggesting that structured approaches to lucid-dream induction could provide an entry point for engaging with contemplative sleep practices. In contrast, the monastic participants were deeply familiar with Chenrezig through waking practices but had limited prior experience with lucid dreaming. Indeed, it can be challenging to engage in spiritual visualization in a dream without training or experience with lucid dreaming. The lucid dream reported by the Tibetan monastic participant in the study does not include an attempt at conjuring Chenrezig, likely because the participant did not remember the task during the lucid dream. Nevertheless, it is notable that this was the participant’s first reported lucid dream and it occurred one night after experiencing the experimental protocol.

Our findings align with growing interest in the use of lucid dreaming for spiritual and transpersonal experiences. Such dreams may serve as a platform for intentionally engaging with symbolic or sacred imagery, including divine beings, especially when supported by induction techniques that harness intention and pre-sleep visualization [9]. While deity visualization is only one of many practices in dream yoga, it plays a central role in tantric Buddhist traditions [7,8] and may offer a unique opportunity to examine interactions between intentional imagery and dream states.

Importantly, this study was both a scientific investigation and a didactic learning experience for monastic scholars. While the goals of training and experimentation were complementary, the limited empirical evidence from this study should be interpreted cautiously. This project constituted one step in advancing the vision enunciated by His Holiness the 14th Dalai Lama of integrating contemplative wisdom into scientific research through authentic cross-cultural partnership [42]. The monastic team conducted the study and contributed to the experimental design together with non-monastic cognitive neuroscientists, and some also participated as subjects. This study was also among the first to adapt Targeted Lucidity Reactivation (TLR) techniques to a Tibetan contemplative framework, pairing modern lucid-dream induction tools with a variant of a traditional deity-visualization practice. After the internship, the monastic scholars led the construction of a new sleep laboratory located in the Drepung Loseling Meditation and Science Center in Mundgod, India. Using their knowledge and skills gained during the internship, they then began conducting studies extending the present results and aiming to further test the combination of western and Buddhist lucid-dreaming techniques for novices.

Limitations of the present study include the small sample size, high variability in participants’ prior lucid-dreaming experience, and the inherent challenge of reliably directing dream content toward specific imagery or narrative themes. The strongest result came from a previously studied frequent lucid dreamer, making it difficult to disentangle individual ability from the intervention’s effect. The monastic participant’s lucid dream reported the night after the lab session occurred outside laboratory monitoring and should therefore be treated as anecdotal rather than evidence of an intervention effect. Given the spiritual and cultural framing of the task, expectancy effects and demand characteristics may have influenced participants’ dream reports. Future studies should incorporate blinding and control conditions to reduce these biases where feasible. Nonetheless, this result opens new directions for investigating the intersection of spirituality and lucid dreaming.

Follow-up studies may benefit from incorporating longer-term training in both lucid dreaming and dream yoga, as well as including expert practitioners. Future studies could aim to refine induction methods by isolating the contributions of individual components, such as the role of sensory cues in increasing lucid-dream frequency and exploring the psychological or emotional effects of deity visualization in lucid dreams. These next steps would build on the foundation laid here by deepening the scientific investigation of dream-based spiritual practice and exploring how ancient contemplative traditions might inform modern understanding of the dreaming mind.

## 5. Conclusions

This proof-of-concept study illustrates how contemplative visualization practices can be paired with laboratory methods for inducing lucid dreams. A key strength of this study was its cross-cultural collaborative framework, in which monastic scholars contributed to the design, implementation, and interpretation of the project. The lucid dream reported in the laboratory referenced an auditory cue presented during REM sleep, highlighting how auditory reminders may help guide lucid dreamers towards completing specific tasks. These results support the feasibility of adapting Targeted Lucidity Reactivation (TLR) within a contemplative context to support compassion cultivation. These findings should be interpreted cautiously given the small sample, heterogeneity in participants’ prior lucid-dreaming experience, and multiple induction elements which may have supported lucid dreaming. Future studies using larger samples and blinded control conditions should test the effects of auditory cue relevance and contemplative training level on lucid dreaming and compassion cultivation. This approach opens a promising path for contemplative neuroscience research on how compassion can be intentionally cultivated during sleep.

## Figures and Tables

**Table 1:** Quantitative summary of cues presented during REM sleep and dream reports collected for each participant.

Participant	Gender	Age	Overnight Sessions	3-Beeps Cues Presented	Chenrezig Cues Presented	Dream Reports Collected
1	Female	40	2	28	3	4
2	Male	39	1	14	0	3
3	Male	44	2	24	16	7
4	Male	43	1	7	0	2
5	Male	23	1	42	2	4

## Data Availability

The original data presented in the study are openly available on OSF at https://osf.io/zu7he/ (accessed on 3 March 2026).

## Abbreviations

The following abbreviations are used in this manuscript:

TLR	Targeted Lucidity Reactivation
ETSI	Emory Tibet Science Initiative
REM	Rapid Eye Movement
